# First person – Sneh Harsh

**DOI:** 10.1242/dmm.044826

**Published:** 2020-04-30

## Abstract

First Person is a series of interviews with the first authors of a selection of papers published in Disease Models & Mechanisms, helping early-career researchers promote themselves alongside their papers. Sneh Harsh is first author on ‘Zika virus non-structural protein NS4A restricts eye growth in *Drosophila* through regulation of JAK/STAT signaling’, published in DMM. Sneh conducted the research described in this article while a postdoctoral researcher in Ioannis Eleftherianos's lab at The George Washington University, Washington, DC, USA. She is now a postdoctoral researcher in the lab of Erika Bach at NYU Langone Health, New York, NY, USA, investigating host pathophysiology upon Zika virus infection using *Drosophila* developing organs as the model system.


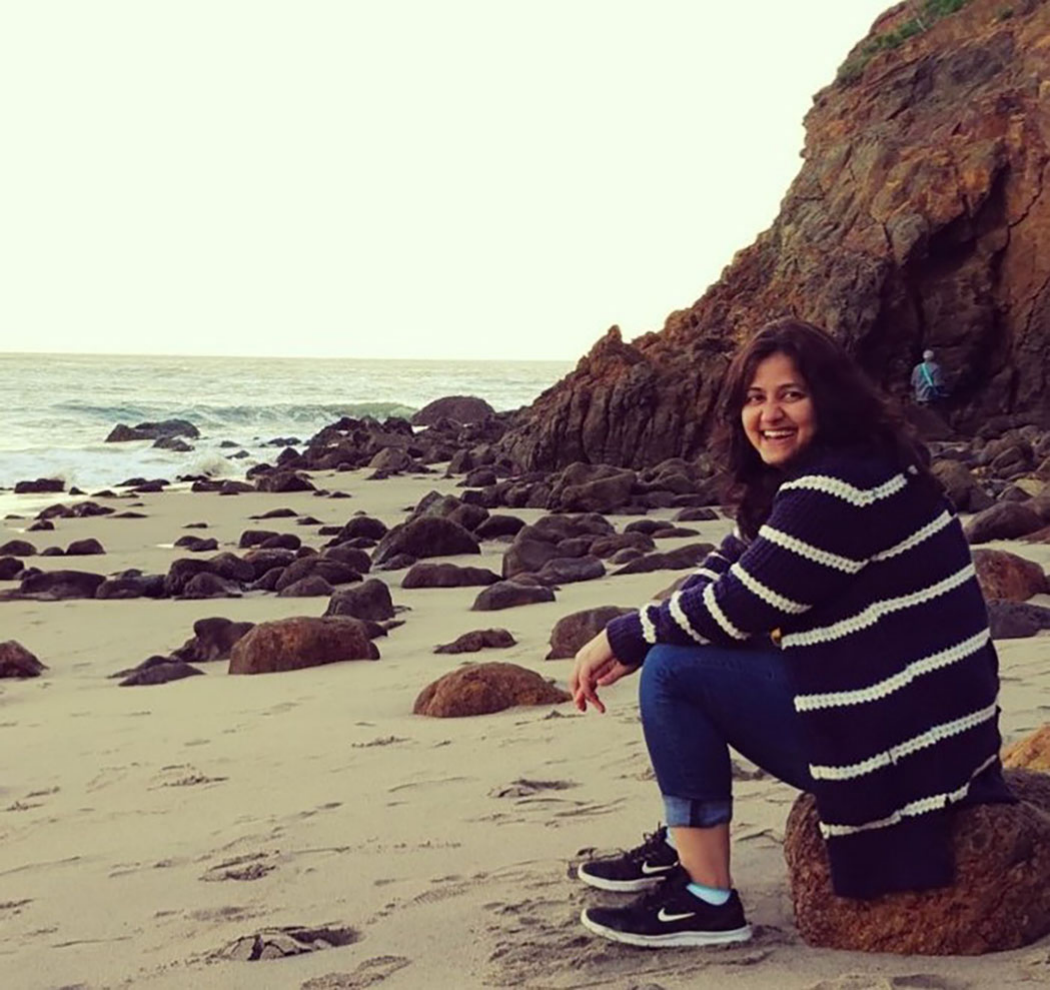


**Sneh Harsh**

**How would you explain the main findings of your paper to non-scientific family and friends?**

Zika virus (ZIKV), belonging to the same family as that of dengue or West Nile virus, has emerged as a global pathogen. Though mostly asymptomatic, its infection in pregnant woman is associated with microcephaly or retarded growth of brain of the newborn child. Being an acellular organism, virus propagation depends on its interaction with the host; a successful interaction results in propagation of virus and, in turn, debilitating outcome for the host, which in turn starts exhibiting severe forms of pathologies. In order to understand the pathogenic mechanism of ZIKV infection, we have used *Drosophila* adults and larvae (developing stage) as the model system. We infected *Drosophila* adults with one of the most pathogenic strains of ZIKV and examined the whole transcriptional profile in the infected host. Based on the analysis of the transcriptional profile and the underlying genetic perturbations, we then switched to the developing larval stage to examine the effect of ZIKV on different developing organs and to confirm if the resulting pathogenesis could indeed be attributed to the same set of genetic perturbations. We found that ZIKV infection results in retarded growth of the developing eye and wing. We further linked the retarded growth with the perturbations of the signaling pathways conserved and known to regulate crucial developmental events in the mammalian system. Therefore, we think this *Drosophila* model for host-ZIKV interaction has the potential to provide useful insights to the community.

**What are the potential implications of these results for your field of research?**

While the replication machinery of viruses has been studied extensively, how the virus interacts with the host and how this interaction dictates the final outcome has not been dealt with extensively. We believe that understanding the host-virus interaction is as critical as understanding the replication machinery of the virus, especially from a therapeutics point of view. Our findings implicating the perturbation of conserved signaling pathways upon ZIKV infection further signify the importance of host pathogenesis in an overall understanding of the host-virus interaction. These findings not only prove the efficacy of *Drosophila* to model viral infections, but also show that the reason that viral infections are so difficult to tackle is because they perturb some of the crucial events in the host development. We believe that these findings will boost host-virus research, especially from the perspective of host pathogenesis and not merely the viral replication machinery.

**What are the main advantages and drawbacks of the model system you have used as it relates to the disease you are investigating?**

*Drosophila* has been an excellent system to model viral infections. The main advantage of *Drosophila* in the context of viral infections is the genetic tools available and the ease with which you can knock out or overexpress any gene of interest in a cell- and time-specific manner. Furthermore, the conservation of most of the *Drosophila* signaling pathways in the mammalian system enhances the overall significance and impact of the study.

However, the main drawback is that ZIKV is not a natural virus of *Drosophila* and *Drosophila* is not a natural carrier of this virus, so the effect it shows in *Drosophila* needs to be taken with a pinch of salt when it comes to its applicability to the mammalian system.

**What has surprised you the most while conducting your research?**

I have been working on *Drosophila* for some time now, so I am familiar with the efficiency of this organism in modeling diseases like cancer. However, the fact that a non-natural virus, which to date is only known to be causing infection in mammals and carried by mosquitoes, can cause such a drastic host pathology and perturb some of the most crucial signaling pathways in an artificial host like *Drosophila* has really surprised and amazed me!

**Figure:**
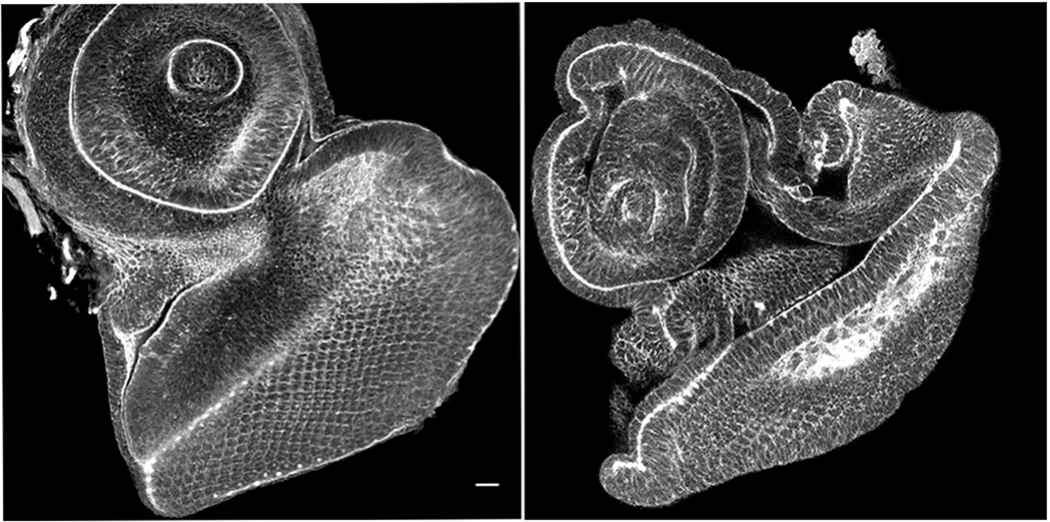
***Drosophila* larval eye imaginal epithelia carrying overexpression of ZIKV NS4A (right) shows significant reduction in size compared to the control eye imaginal epithelia (left).**

**Describe what you think is the most significant challenge impacting your research at this time and how will this be addressed over the next 10 years?**

A major challenge in Zika virus infection is to understand the stages of the infection including the tropism and the dynamics with the host. Until now, there is no clear understanding of how ZIKV enters inside the host cells, how it facilitates its own replication once inside the host and how it hijacks the host machinery and affects the host growth. For a successful infection, one of the prerequisites is to evade the host immunity. With the increasing advent of new viral infections, it becomes more imperative to identify the host immune responses triggered upon these viral infections and the mechanism for successful evasion from this response. In particular, it would be interesting to examine immune priming, where the host immune response has been primed for other similar viruses like dengue, and then examine the effect on the host immune response when encountering ZIKV infection. With the emergence of new studies increasingly implicating lipid droplets in several infections, I believe that host lipid droplets could be instrumental in overall host-virus interaction and the outcome thereof. Lipid profiling of an infected host and understanding how the host lipid composition affects the viral infection would play a major role in overall understanding of host-virus interaction.

**What changes do you think could improve the professional lives of early-career scientists?**

I think communication is a key aspect of science. Developing initiatives at the university level to enhance scientific communication skills would definitely improve the professional lives of early-career scientists. Specifically, the ability to explain science to a diverse audience and advocate the importance of science to the general public should be the main aspect of such training, which in turn will also be instrumental in connecting science with the society.

“[…] the fact that a non-natural virus […] can cause such a drastic host pathology and perturb some of the most crucial signaling pathways in an artificial host like *Drosophila* has really surprised and amazed me!”

**What's next for you?**

I am currently a postdoctoral fellow at NYU Langone Health working in the lab of Dr Erika Bach. The main focus of the lab is to understand the dynamics of stem cells during development and special circumstances such as stress or genetic aberrations using *Drosophila* testis as the model system. I am excited to learn these new aspects of *Drosophila* biology, which I would like to explore in the context of cancerous systems. In the near future, I would like to open my own research lab, which will focus on different aspects of cancer development or progression, and also incorporate my experience and expertise on viral studies and stem cell behavior.

## References

[DMM044826C1] Harsh S., Fu Y., Kenney E., Han Z. and Eleftherianos I. (2020). Zika virus non-structural protein NS4A restricts eye growth in *Drosophila* through regulation of JAK/STAT signaling. *Dis. Model. Mech.* 13, dmm040816 10.1242/dmm.040816 PMC7197722 32152180

